# Preparation and Evaluation of Anti-Fatigue Effects of Sea Buckthorn–Wolfberry Compound Coffee

**DOI:** 10.3390/foods14162818

**Published:** 2025-08-14

**Authors:** Yuxian Chen, Lili Zhao, Qinghui Wang, Xuhai Yang, Jun Wang

**Affiliations:** 1 College of Food Science and Engineering, Northwest A&F University, Yangling, Xianyang 712100, China; yuxianchen24@m.fudan.edu.cn (Y.C.); lili.zhao@nwafu.edu.cn (L.Z.); 2 School of Public Health, Fudan University/Key Laboratory of Public Health Safety, Ministry of Education, Shanghai 200032, China; 3 Agricultural Mechanization Institute, Xinjiang Academy of Agricultural Sciences, Ürümqi 830091, China; wangqh1201@126.com; 4 College of Mechanical and Electrical Engineering, Shihezi University, Shihezi 832003, China; yxh_513@shzu.edu.cn

**Keywords:** sea buckthorn, wolfberry, compound coffee, antioxidation, anti-fatigue

## Abstract

In this study, a sea buckthorn–wolfberry compound coffee (SWCC) solid beverage was formulated and evaluated based on sensory scores, dispersibility, and water solubility. The optimal formulation consisted of 9% sea buckthorn powder, 16% wolfberry powder, 65% coffee powder, 8% sugar, 1.25% microcrystalline cellulose, 0.5% sodium bicarbonate, and 0.25% tricalcium phosphate. The SWCC contained 18.75 ± 0.43 mg RE/g total flavonoids and 4.60 ± 0.04 mg GAE/g total phenols, demonstrating superior *in vitro* antioxidant activity compared to the raw sea buckthorn or wolfberry powders, with a 90.21 ± 0.15% DPPH radical scavenging rate, 90.56 ± 0.35% ABTS radical scavenging rate, and 6.64 ± 0.03 mg Trolox/g ferric-reducing power. *In vivo* experiments showed that specific doses (1.25–5.00 g/kg·BW/day) of SWCC exhibited significant physical fatigue-relieving and antioxidant effects, significantly extending loaded swimming time, reducing BLA accumulation, increasing LG reserves, enhancing SOD activity, and lowering MDA levels in serum. Overall, our findings offer both theoretical and practical insights for utilizing medicinal and edible resources in functional food development, meeting the growing demand for healthy and diverse food options, and contributing significantly to the advancement of public nutrition and the healthy food industry.

## 1. Introduction

Fatigue is a primary symptom of sub-health in humans. Prolonged fatigue can harm both physical and mental health, disrupt normal life, and predispose individuals to various diseases [1]. Based on physiological and biochemical changes in the body, fatigue can be classified into physical and psychological categories, which are interrelated. Some studies have indicated that muscle fatigue can exacerbate mental fatigue, highlighting the importance of preventing physical fatigue to alleviate overall exhaustion [2]. There are two primary methods to alleviate fatigue. One method involves physical interventions such as acupuncture and massage, which are limited by the need for professional equipment and skilled personnel. The other method involves chemical interventions such as medication, injections, and dietary or nutritional supplements; however, some medications may cause side effects [3].

Currently, resources that serve both medicinal and nutritional purposes are widely utilized in anti-fatigue research and applications [4]. Traditional Chinese medicine and its derived foods primarily alleviate fatigue by regulating energy depletion, harmful metabolite production, oxidative stress response, intestinal flora imbalance, inflammation, and abnormal neurotransmitter levels [5]. Sea buckthorn (*Hippophae rhamnoides* L.) and wolfberry (*Lycium barbarum* L.) are plants with dual medicinal and nutritional uses, showing potential for application in anti-fatigue functional foods. Numerous studies have demonstrated that flavonoids, polyphenols, and other active ingredients isolated and purified from sea buckthorn or wolfberry exhibit antioxidation and anti-fatigue effects in both *in vitro* chemical experiments and *in vivo* animal studies [6,7]. Coffee (*Coffea arabica* L.), which contains caffeine, chlorogenic acid, flavonoids, terpenoids, and other bioactive components, is one of the three major beverages consumed globally. Moderate consumption can provide refreshing and antioxidant benefits. Coffee can effectively scavenge free radicals and has strong iron-reducing capabilities. Its antioxidant effects are strongly correlated with the total flavonoid and phenolic acid content, which accumulate during cultivation and processing [8].

Nowadays, global consumption of functional foods and beverages has risen due to the WHO’s promotion of their health benefits and increasing consumer focus on nutrition. The functional drinks market is especially vibrant, with innovative products that enhance the bioavailability of bioactive compounds, offering health benefits and containing essential nutrients from various sources [9]. Therefore, health benefits, not just sensory appeal, are key drivers of consumer choices for beverages, leading to a market growth that reflects a demand for healthier options [10]. In this study, sea buckthorn, wolfberry, and coffee powder were used as the main raw materials to prepare a sea buckthorn–wolfberry compound coffee (SWCC) solid beverage through optimization. The sensory characteristics, physical and chemical properties, and nutritional functions of SWCC were evaluated. Additionally, its anti-fatigue effects were investigated through animal experiments, providing both theoretical and practical references for the development of anti-fatigue foods and innovative coffee products.

## 2. Materials and Methods

### 2.1. Chemicals and Reagents

Fresh sea buckthorn fruits were harvested from Xinjiang Province, China, and processed into sea buckthorn powder by Bozhou Huazhitang Biotechnology Co., Ltd. (Bozhou, China). Fresh wolfberry fruits were sourced from Qinghai Province, China, and processed into wolfberry powder by Taizhou Lianheng Food Co., Ltd. (Taizhou, China). The two above mentioned powders were both produced through pulping, lyophilization, and pulverization. Coffee powder was purchased from Old Town White Coffee Co., Ltd., Ipoh, Malaysia. All food additives were food-grade products available on the market. The other chemical reagents used were of the highest commercially available purity.

### 2.2. Preparation of SWCC

The SWCC solid beverage consisted of sea buckthorn powder; wolfberry powder; coffee powder; white granulated sugar (Taikoo Sugar Co., Ltd., Hong Kong, China); and a food additive mixture containing microcrystalline cellulose (Huzhou City Linghu Xinwang Chemical Co., Ltd., Huzhou, China), sodium bicarbonate (Tianjin Bohua Yongli Chemical Industry Co., Ltd., Tianjin, China), and tricalcium phosphate (Lianyungang Kede Food Ingredients Co., Ltd., Lianyungang, China) (*w*/*w*/*w* = 5:2:1). The proportion of food additives, where microcrystalline cellulose acted as an anti-caking agent, sodium bicarbonate functioned as a pH-regulating agent, and tricalcium phosphate served as a stabilizer, was determined in accordance with the *Chinese Standards for Food Additives* (GB 2760-2024 [11]) and preliminary experiments. These seven ingredients were blended and then sieved through an 80-mesh screen to obtain the SWCC powder. Based on single-factor and orthogonal experiments, the optimal SWCC formula was determined using key evaluation criteria such as sensory scores, dispersibility, and water solubility.

#### 2.2.1. Single Factor Tests

##### Determination of the Addition Amount of Sea Buckthorn Powder

Based on preliminary experiments, the amounts of wolfberry powder, sugar, and additive mixture were fixed at 13%, 10%, and 2% of the total SWCC composition, respectively, while sea buckthorn powder was added at varying levels of 3%, 6%, 9%, 12%, and 15%.

##### Determination of the Addition Amount of Wolfberry Powder

Based on preliminary experiments, the amounts of sea buckthorn powder, sugar, and additive mixture were fixed at 9%, 14%, and 2% of the total SWCC composition, respectively, while wolfberry powder was added at varying levels of 10%, 13%, 16%, 19%, and 22%.

#### 2.2.2. Orthogonal Tests

Based on single-factor tests, the amounts of sea buckthorn powder, wolfberry powder, coffee powder, and additive mixture were chosen as test factors, and interactions between these factors were not considered. A 4-factor, 3-level experimental design was employed to optimize the SWCC formula. The test factors and their levels are presented in Table 1.

#### 2.2.3. Comprehensive Evaluation of SWCC

##### The Sensory Test

SWCC was brewed at 90 °C in various proportions following both single-factor and orthogonal tests (*w*/*v* = 1:5). Five core evaluation criteria were selected, namely color, aroma, taste, texture morphology, and brewing performance, with each criterion assigned 20 points from a total of 100. The optimal characteristics for each criterion were as follows: dark brown and uniform color, distinctive and harmonious aroma, well-balanced taste, fine and dry powder with high fluidity, and rapid dissolution without flocs or agglomeration [12]. Thirty professionally trained volunteers who met the sensory evaluation requirements were selected as the evaluation group.

##### The Dispersion Test

A 2.00 g sample was mixed with 10 mL of distilled water at 90 °C using a magnetic stirrer (JB-11, Shanghai Leici Biotechnology Co., Ltd., Shanghai, China) to measure the time needed for complete powder dispersion [13].

##### The Water Solubility Test

A 1.00 g sample was combined with 5 mL of distilled water at 90 °C in a 25 mL beaker. After stirring, the solution was centrifuged at 3000 rpm for 10 min at 4 °C, using a centrifuge (TG16, Changsha Yingtai Instrument Co., Ltd., Changsha, China), and 2 mL of the supernatant was transferred into a pre-weighed conical flask. The flask was then placed in an oven (WGL-230B, Tianjin Taist Instrument Co., Ltd., Tianjin, China) at 105 ± 5 °C until it reached a constant weight, and the powder’s solubility was calculated using Equation (1) [14].

(1) $$\mathrm{solubility}=\frac{2.5\;\times \;(m_{2}-m_{0})}{m_{1}}\times 100$$

where m_0_, m_1_, and m_2_ represent the weight of the conical flask (g), the powder (g), and the total weight of the flask with dry powder (g), respectively. Water solubility was defined as the mass of SWCC powder dissolved in 100 g of water at 90 °C.

### 2.3. Evaluation of Nutritional Quality and Antioxidant Activity of SWCC

#### 2.3.1. Determination of Physical Properties

##### Determination of Fluidity

The fluidity of the powders was comprehensively analyzed using the repose angle and slide angle.

Determination of the repose angle: A 90 mm diameter glass funnel was mounted on a retort stand, ensuring the funnel tip was positioned 6 cm above a horizontal surface. A glass plate was positioned on the surface to collect the powder falling freely from the funnel. A total of 5 g of sample was gently poured into the funnel, allowing it to fall naturally and form a cone on the glass plate. The repose angle was then calculated using Equation (2) [15]:

(2) $$\tan\alpha =\frac{h}{r}$$

where α, h, and r represent the repose angle (°), the height of the powder cone (cm), and the circular radius of the bottom surface of the powder cone (cm).

Determination of the slide angle: 2 g of sample was placed on one end of a glass plate (10 cm × 10 cm). This end of the glass plate was slowly raised until approximately 10% of the initial powder remained. The angle between the glass plate and the horizontal surface at this point was recorded as the slide angle [16].

##### Determination of Clarity

A 1% (*m*/*v*) solution was prepared using water heated to 90 °C. After being cooled to room temperature (25 °C), the transmittance of the sample solution was measured at a wavelength of 680 nm (UV-1800, Shimadzu Co., Ltd., Kyoto, Japan), using distilled water as the reference standard [17].

##### Determination of Soluble Solids Content (SSC)

A total of 1.00 g of powder was added to 5 mL of distilled water heated to 90 °C and magnetically stirred for 1 min to ensure complete dissolution. The soluble solids content in each sample solution (*m*/*v* = 1:5) was determined using a refractometer (PAL-1, ATAGO Co., Ltd., Tokyo, Japan) at 25 ± 2 °C [14].

##### Determination of Chromaticity

The chromaticity of the powder and its solution (*w*/*v* = 1:5) was measured using a colorimeter (CS-820, Hangzhou Color Spectrum Technology Co., Ltd., Hangzhou, China) at room temperature (25 ± 1 °C). According to the color system of the International Commission on Illumination (CIE), a CIE standard illuminant D65 and a 10° observer were used to determine the parameters L^*^ (lightness, 0–100), a^*^ (red–green, −60 to +60), and b^*^ (yellow–blue, −60 to +60) [18].

#### 2.3.2. Evaluation of Nutritional Quality of Compound Coffee

##### Preparation of the Extract

First, 1.00 g of powder was accurately weighed and mixed with 95% ethanol (*v*/*v*) to a final volume of 25 mL. The mixture was then ultrasonically extracted at 50 °C and 40 kHz for 20 min (JM-30D-40, Skymen Cleaning Equipment Shenzhen Co., Ltd., Shenzhen, China) [19]. After centrifugation at 8000 rpm for 30 min at 4 °C, the supernatant was collected for further analysis.

##### Determination of Total Flavonoid Content (TFC)

The total phenolic content was determined using the aluminum trichloride colorimetric method, measured at 510 nm, with rutin as the reference standard, and expressed as milligrams of rutin equivalents (mg RE) per gram of sample [20].

##### Determination of Total Phenol Content (TPC)

The total phenolic content was determined using the Folin–Ciocâlteu method, measured at 765 nm with gallic acid as the reference standard and expressed as milligrams of gallic acid equivalents (mg GAE) per gram of sample [6].

#### 2.3.3. Determination of Antioxidant Capacity of SWCC

##### Preparation of Extract

The steps were the same as those in Section 2.3.2’s “Preparation of the Extract”.

##### Determination of DPPH Radical Scavenging Rate

The DPPH radical scavenging rate was measured using the method described by Ciganović et al. [21], with minor adjustments. The absorbance of each reaction solution was measured at 517 nm, using 95% ethanol as the reference, and the results were calculated using Equation (3):

(3) $$\mathrm{DPPH}\;\mathrm{radical}\;\mathrm{scavenging}\;\mathrm{rate}/\%\;=\;\frac{A_{1}-A_{2}}{A_{1}}\times 100$$

where A_1_ and A_2_ are the absorbance of ethanol control group and sample determination group.

##### Determination of ABTS Radical Scavenging Rate

The ABTS radical scavenging rate was measured using the method from Turcov et al. [22], with minor adjustments. The absorbance of each reaction solution was measured at 734 nm, using 95% ethanol as the reference, and the results were calculated using Equation (4):

(4) $$\mathrm{ABTS}\;\mathrm{radical}\;\mathrm{scavenging}\;\mathrm{rate}/\%\;=\;\frac{A_{1}-A_{2}}{A_{1}}\times 100$$

where A_1_ and A_2_ are the absorbance of the ethanol control group and sample determination group.

##### Determination of Ferric-Reducing Antioxidant Power (FRAP)

The ferric-reducing antioxidant power was measured using the method described by Navoda et al. [23], with minor adjustments. The absorbance of each reaction solution was measured at 593 nm, using Trolox as the reference, and the results were expressed as milligrams of Trolox equivalents (mg TE) per gram of sample.

### 2.4. Evaluation of Anti-Fatigue Function of SWCC

#### 2.4.1. Experimental Animals and Treatments

Sixty male SPF Institute of Cancer Research (ICR) mice (28 ± 2 g, 6 weeks) were purchased from Chengdu Dashuo Experimental Animal Co., Ltd., Chengdu, China. The mice were housed under a 12 h light/dark cycle, with a temperature range of 22–26 °C and 40–70% relative humidity, and provided with free access to a standard diet (SPF-F02-001, SiPeiFu Biotechnology Co., Ltd., Beijing, China) and water in a barrier facility. The animal experiments were approved by the Experimental Animal Center Subcommittee of Northwest A&F University (Yangling, China, Protocol No. XITULAC 2014-1035) and were performed in accordance with the *Chinese Guide for the Care and Use of Laboratory Animals*.

#### 2.4.2. Animal Grouping and Administration

After a 7-day acclimation period, all mice were randomly assigned to five groups (12 ICR mice per group) according to the *Technical Guidelines for Functional Testing and Evaluation of Health Foods* (2023 Edition): (1) negative control group (NC), treated with water; (2) positive control group (PC), treated with normal coffee from Old Town White Coffee Co., Ltd., Malaysia (1.25 g/kg·BW/day); (3) low-dose group (L), treated with SWCC (1.25 g/kg·BW/day); (4) medium-dose group (M), treated with SWCC (2.50 g/kg·BW/day); and (5) high-dose group (H), treated with SWCC (5.00 g/kg·BW/day). The doses for the L, M, and H groups were calculated based on an estimated daily intake of approximately 8, 16, and 32 g of coffee powder for an average adult, which conformed to recommended amount of coffee intake from both the *Dietary Guidelines for Chinese Residents* (2022) and *The Dietary Guidelines for Americans* (2015). The corresponding dose of each intervention was administered intragastrically at 0.1 mL/10 g·BW daily for 4 weeks, between 9:00 a.m. and 11:00 a.m. [24].

The preparation of PC and each dose group before daily intragastric administration was as follows: ordinary coffee powder or SWCC powder was dissolved in 90 °C hot water at the designated concentration for each group, stirred magnetically for 2 min while heating, and then cooled to room temperature (25 ± 1 °C). After the final administration and a 30 min rest period, the mice in each group were divided into two subgroups: A and B (6 ICR mice per subgroup). Subgroup A was used for the forced swimming test, while subgroup B was used for the determination of physiological and biochemical parameters [25].

#### 2.4.3. Determination of Body Weight and Organ Index of Mice

The body weight of the mice was measured before the experiment began and again after it started, with weekly recordings throughout the study. At the end of the experiment, the mice were first given tribromoethanol (Beijing Jitian Bio-Technology Co., Ltd., Beijing, China) at 0.15 mL/10 g·BW via intraperitoneal injection to perform the euthanasia procedure. Then, they were dissected, and the hearts, livers, spleens, lungs, and kidneys were collected. These organs were thoroughly rinsed with 0.9% saline to remove blood, connective tissue, and fat. Excess moisture was removed using filter paper, and then their weights were measured accurately. The organ index was calculated using Equation (5) [20]:

(5) $$\mathrm{organ}\;\mathrm{index}/\%\;=\frac{m_{1}}{m_{0}}\times 100\%$$

where m_0_ and m_1_ are the weight of the mouse and the specific organ.

#### 2.4.4. The Weight-Bearing Swimming Test

The weight-bearing swimming test was conducted following a previously established method, with minor modifications [26]. Mice in subgroup A of each group were fitted with galvanized iron wire attached to their tails, weighing 5% of their body weight, and then placed in a swimming pool (55 cm × 40 cm × 35 cm) filled with 30 cm of water at 25 ± 1 °C. If the mouse’s head remained submerged for more than 7 s without resurfacing, it was considered physically exhausted. The time from the start of the test to exhaustion was recorded precisely to assess physical fatigue.

#### 2.4.5. Determination of Fatigue-Related Biochemical Indicators

The fatigue model was established using forced swimming in subgroup B of each group, and biochemical markers related to physical fatigue were measured [25]. Thirty minutes after the final administration, the mice were made to swim unburdened for 30 min in the pool, which was maintained at 30 ± 1 °C; after swimming, they were towel-dried and allowed to rest under a heat lamp for 30 min to stay dry. Then, all mice were anesthetized via intraperitoneal injection of tribromoethanol (0.15 mL/10 g·BW) and sacrificed to collect blood samples from the eyeballs, as well as organ tissues. Serum was obtained through centrifugation at 3500 rpm and 4 °C for 10 min and stored at −80 °C for later experiments. The organs were weighed, and organ indices were calculated, with the liver specifically being rinsed with 0.01 M PBS buffer, dried with filter paper, placed in a freezer tube, and stored at −80 °C [27]. Using kits from Nanjing Jiancheng Biotechnology Institute (Nanjing, China), biochemical markers related to fatigue and oxidative stress, such as blood lactic acid (BLA), blood urea nitrogen (BUN), liver glycogen (LG), superoxide dismutase (SOD), and malondialdehyde (MDA), were measured separately.

### 2.5. Statistical Analysis

All experiments were performed in triplicate, with data presented as the mean ± standard deviation (M ± SD). An orthogonal experimental design was implemented using Minitab software (version 19.1). IBM SPSS Statistics (version 26) was used to analyze significance levels. Data normality was assessed using the Shapiro–Wilk test, and analysis of variance (ANOVA) was performed using Duncan’s test. Data visualization was conducted using Origin software (version 2024).

## 3. Results and Discussions

### 3.1. Preparation of SWCC

#### 3.1.1. Properties of Raw Material Powder

**Figure 1:**
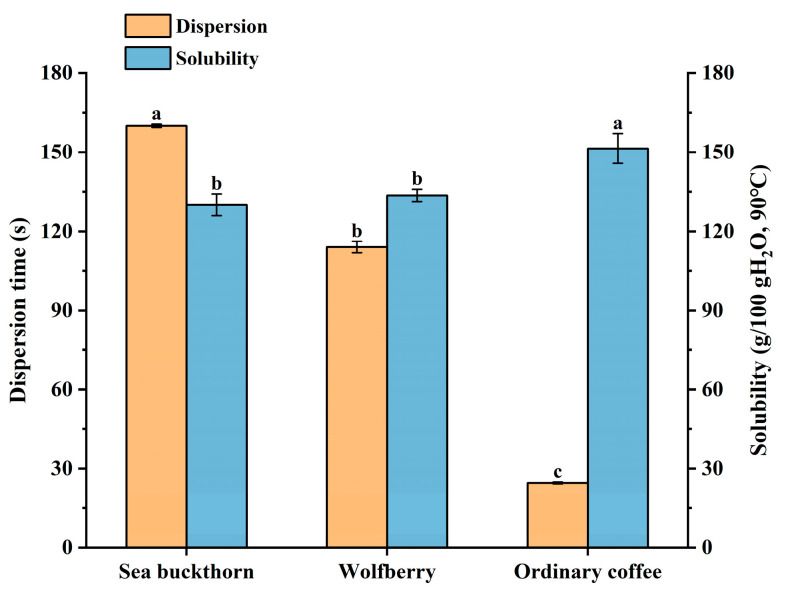
Properties of raw material powders of SWCC. Note: Different letters represent mean differ at *p* < 0.05.

Dispersion and water solubility are critical indicators for assessing the brewing performance of solid beverages [28]. Dispersion indicates the rate at which the powder dissolves, with shorter dispersion times suggesting faster, more uniform diffusion and minimal agglomeration. Water solubility defines the solubility of the powder and depends on the properties of the substances involved (solvent and solute), as well as external factors (temperature, pressure, solvent type, etc.). The primary ingredients of SWCC are sea buckthorn powder, wolfberry powder, and coffee powder. Of these, coffee powder exhibits the highest dispersibility and solubility (Figure 1). Thus, during the preparation of SWCC, it is advisable to reduce the proportion of fruit powders and increase the amount of coffee powder, while maintaining sensory quality, to ensure optimal brewing properties. Moreover, powders derived from fruits and vegetables are utilized in the beverage sector to enhance nutritional content, serve as flavor enhancers in products like ice cream and yogurt, and act as natural colorants. While numerous studies have examined instant powders and drinks, the development of powdered beverages that incorporate a variety of bioactive compounds faces constraints due to their unique chemical profiles and the need for sensory and physicochemical assessments [29]. Therefore, the solid beverage in this study was formulated primarily with coffee powder, while sea buckthorn and wolfberry powders were incorporated to enhance both the sensory appeal and nutritional value through their synergistic effects.

#### 3.1.2. Single Factor Test Results

**Figure 2:**
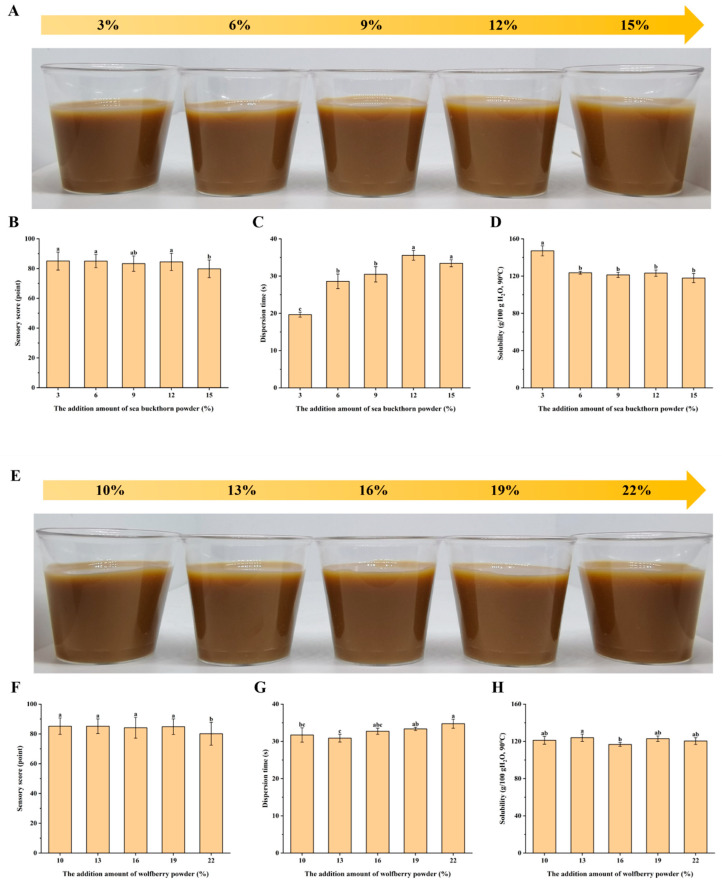
Effect of sea buckthorn/wolfberry powder addition on properties of SWCC. (**A**) Compound coffee solution with different amounts of sea buckthorn powder added. (**B**) The effect of sea buckthorn powder supplemental level on sensory scores of SWCC. (**C**) The effect of sea buckthorn powder supplemental level on dispersity of SWCC. (**D**) Effect of sea buckthorn powder addition on solubility of SWCC. (**E**) Compound coffee solution with different amounts of wolfberry powder added. (**F**) The effect of wolfberry powder supplemental level on sensory scores of SWCC. (**G**) The effect of wolfberry powder supplemental level on dispersity of SWCC. (**H**) Effect of wolfberry powder addition on solubility of SWCC. Note: Different letters represent mean differences at *p* < 0.05.

Based on comprehensive evaluations of sensory quality, dispersibility, and water solubility, the optimal addition levels of sea buckthorn and wolfberry powders were determined to be 3–12% and 10–19%, respectively (Figure 2).

#### 3.1.3. Orthogonal Tests

Based on the results of ANOVA, the addition of sea buckthorn powder, wolfberry powder, coffee powder, and the additive mixture significantly impacted (*p* < 0.01) the sensory score and dispersibility of SWCC. Additionally, the addition of sea buckthorn powder, wolfberry powder, and the additive mixture also significantly affected (*p* < 0.01) the water solubility of SWCC. The optimal SWCC formula was identified using the comprehensive balance method, with the optimal additive levels being 9% sea buckthorn powder, 16% wolfberry powder, 65% coffee powder, 8% sugar, 1.25% microcrystalline cellulose, 0.5% sodium bicarbonate, and 0.25% tricalcium phosphate.

### 3.2. Evaluation of Nutritional Quality and Antioxidant Activity of SWCC

#### 3.2.1. Determination of Physical Properties

##### Determination of Fluidity

**Figure 3:**
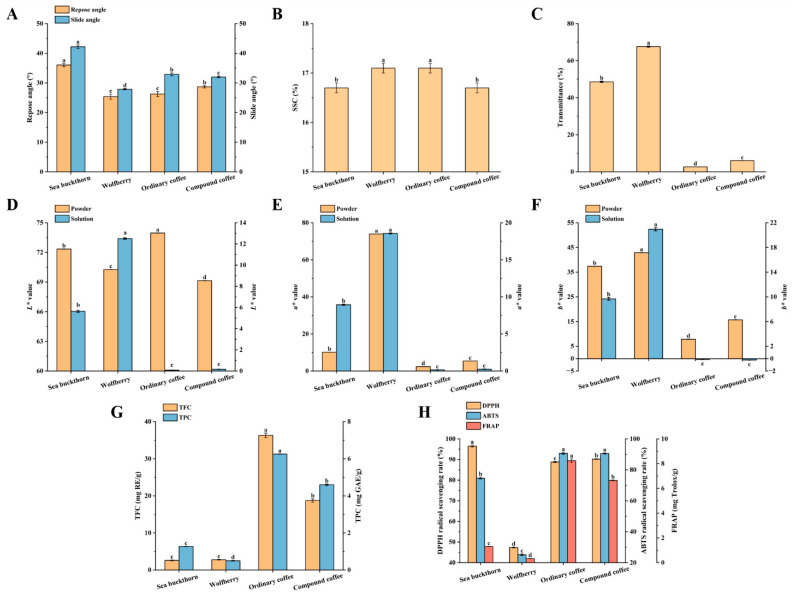
Evaluation of nutritional quality and antioxidant activity of SWCC. (**A**) Flowability of powders. (**B**) Clarity degree of solution. (**C**) Soluble solid content of solution (1:5, *w*/*v*). (**D**) *L* ^*^ value of powders/solution. (**E**) *a* ^*^ value of powders/solution. (**F**) *b* ^*^ value of powders/solution. (**G**) Total flavonoid content and total phenol content in powders. TFC was calculated per gram of powder, measured by rutin, the standard curve: y = 0.0004x − 0.002 (R^2^ = 0.9989); TPC was calculated per gram of powder, measured by gallic acid, the standard curve: y = 0.0071x + 0.0191 (R^2^ = 0.9986). (**H**) Oxidation resistance of powders; FRAP was calculated per gram of powder, measured by Trolox, the standard curve: *y* = 0.0062*x* + 0.0811 (R^2^ = 0.9953). Note: Different letters represent mean differences at *p* < 0.05.

Fluidity is a critical factor in assessing the storage stability of powders, which is inversely correlated with the repose angle and slip angle [16]. Figure 3A shows that sea buckthorn powder had the poorest fluidity, while wolfberry powder had the best, and that the fluidity of SWCC powder, after incorporating fruit powders, was similar to that of ordinary coffee powder.

##### Determination of Clarity

Transmittance is a key metric for measuring the clarity of a solution. Improved solution clarity weakens the blocking effect of suspended particles on light, thereby reducing the solution’s light-absorption capacity [30]. As shown in Figure 3B, the clarity of the 1% (*m*/*v*) solutions for each sample was as follows: wolfberry > sea buckthorn > compound coffee > ordinary coffee, indicating that the clarity of the SWCC solution is significantly improved after combining fruit powders with coffee powder (*p* < 0.05).

##### Determination of SSC

The content of soluble solids is a crucial indicator for measuring the total amount of soluble substances in a solution. Sweetness, determined by soluble sugar content, is a primary evaluation criterion for beverage quality and consumer acceptance. Given that nearly 85% of the soluble solids in many fruit-based drinks are sugars and that obtaining the SSC value is straightforward, SSC is commonly used to represent the solution’s sweetness [31]. As shown in Figure 3C, the SSC of the SWCC solution (16.7 ± 0.1%) was significantly lower than that of ordinary coffee powder (*p* < 0.05), indicating that SWCC contains less sugar.

Inevitably, SWCC contained 8% added sugar to balance the sourness of fruit and vegetable powders with coffee. A South Korean study found that the average sugar content in seven Dalgona lattes (DLs) from Seoul was 12.3 g/100 g, with 32.5 g per serving, and sucrose was the predominant sugar, constituting 64% [32]. Therefore, SWCC had a lower sugar content than these lattes and original coffee in our study. However, beverages notably contribute the most to daily added sugar intake, making sugar reduction in them crucial. Future product development could incorporate strategies like direct sugar reduction, multi-sensory design, use of sweeteners, and sweetness enhancers to align with current preferences for reduced sugar consumption [33].

##### Determination of Chromaticity

Color is a critical intuitive factor for consumers in evaluating the appearance quality of products [18]. When observed at room temperature under natural light, the colors of sea buckthorn, wolfberry, ordinary coffee, and SWCC powders were yellow, orange, brown, and cinnamon, respectively. After infusion with hot water, the sea buckthorn and wolfberry solutions were yellow and orange, respectively, while both SWCC and ordinary coffee solutions appeared similarly brown. Based on colorimeter results (Figure 3D–F), SWCC powder had a lower L^*^ value (69.14 ± 0.01) and higher a^*^ (5.39 ± 0.02) and b^*^ (15.72 ± 0.04) values compared to ordinary coffee powder, attributed to the addition of fruit powders. However, no significant difference in chroma was observed between ordinary coffee and SWCC after brewing (*p* > 0.05), indicating that SWCC exhibited favorable product characteristics.

#### 3.2.2. Evaluation of Nutritional Quality of SWCC

Polyphenolic compounds, encompassing phenolic acids and flavonoids, are important bioactive components widely found in sea buckthorn, wolfberry, and coffee [34,35,36]. Polyphenols exhibit anti-fatigue effects, which are attributed to their strong antioxidant activity [37]. The phenolic hydroxyl groups in phenolic compounds can react with free radicals to form stable semi-quinone free radicals, thereby terminating free radical chain reactions, inhibiting the generation of free radicals, and reducing lipid peroxidation [6]. TFC and TPC are highly correlated with the scavenging ability of DPPH· free radicals and the chelating ability of transition metal ions such as Fe^3+^ and Cu^2+^ [38]. Additionally, the bioactivities of these nutrients may be affected by food processing. For example, ball-milling onion (*Allium cepa* L.) peel powder for 18 h showed increased total phenolic content and antioxidant activity but decreased when the ball-milling time was extended to 24 h [19].

As shown in Figure 3G, TFC and TPC in ordinary coffee powder were the highest, at 36.33 ± 0.61 mg RE/g and 6.26 ± 0.01 mg GAE/g, respectively, and were significantly higher than TFC (18.75 ± 0.43 mg RE/g) and TPC (4.60 ± 0.04 mg GAE/g) in SWCC powder (*p* < 0.05). However, TFC and TPC levels in sea buckthorn and wolfberry powders were low, indicating that the total phenols and flavonoids in SWCC were mainly derived from coffee powder. Notably, the TFC and TPC values of the powders in this study differed from those in other reports, which may be due to variations in raw material types, processing technologies, storage times, and extraction methods. While ethanol extraction was used to quantify SWCC’s TFC and TPC, it is important to note that this method captured a broader range of constituents than those solely soluble in water, including moderately lipophilic compounds that may not be fully extracted in aqueous solutions during consumption [39,40,41].

#### 3.2.3. Determination of Antioxidant Capacity of SWCC

The theory of free radical influence is pivotal in understanding the mechanism of fatigue, suggesting that the abnormal generation of reactive oxygen species (ROS) due to intensive exercise under anaerobic conditions leads to oxidative stress and significantly contributes to muscle fatigue [42]. Numerous reported anti-fatigue foods and agents are antioxidants [43], such as corn [44], *Dendrobium officinale* [45], and *Brassica rapa* L. [25]. An effective approach to explore the activity and mechanism of anti-fatigue substances involves first determining their antioxidant properties in vitro through chemical experiments, followed by evaluating responses to oxidative stress in vivo using animal models [4,26]. As shown in Figure 3H, sea buckthorn powder exhibited the highest DPPH free radical scavenging rate (96.53 ± 0.46%); ordinary coffee powder (90.67 ± 0.58%) and SWCC powder (90.56 ± 0.35%) had the highest ABTS free radical scavenging rates; and ordinary coffee powder demonstrated the strongest FRAP (8.23 ± 0.15 mg Trolox/g). Based on the comprehensive analysis of the three methods, ordinary coffee powder exhibited the strongest antioxidant capacity, followed by SWCC powder, with wolfberry powder being the weakest. This was consistent with the TFC and TPC results, indicating that ordinary coffee powder, which constitutes the largest proportion of SWCC, played a major antioxidant role.

### 3.3. Evaluation of Anti-Fatigue Function of SWCC

#### 3.3.1. Effects of SWCC on Physiological Indexes of Mice

Changes in animal body weight can directly reflect the effects of SWCC on growth [27]. As shown in Table 2, there was no significant difference in body weight among the five groups of mice (*p* > 0.05) during the 4-week gavage period. Body weight increased gradually, but the growth rate slowed over time. No significant difference was observed between the SWCC-treated experimental group and the NC group given distilled water (*p* > 0.05). Additionally, no abnormalities in dietary intake or excretion behavior were observed across the groups, indicating that SWCC of all doses did not negatively affect the growth or development of the mice.

The liver is the primary metabolic and largest detoxification organ in the body. The kidneys are the primary excretory organs. The spleen is the largest lymphoid organ and plays a crucial role in the immune system. The cardiopulmonary system is closely related to athletic performance. Therefore, assessing organ indices can provide indirect insights into the development of animal organs and their ability to cope with external doses [46]. As shown in Table 3, there were no significant differences in liver, kidney, spleen, and heart indices between the SWCC dose groups and the two control groups (*p* > 0.05). The results indicated that SWCC had no significant effect on the normal growth and physiological metabolism of mice, nor did it exhibit any toxic or side effects generally. However, the spleen index in the high-dose (H) group was significantly higher than in the NC and PC groups (*p* < 0.05), indicating that an excessive dose of SWCC may affect the spleen and its related immune functions in mice. Notably, this phenomenon was also observed in functional complex extracts of *Rhodiola crenulata*, *Panax quinquefolius*, and *Astragalus membranaceus*, where the spleen index and T- and B-lymphocyte proliferation were significantly increased in mice treated with the extract at 200 mg/kg (high dose group) compared with that of the control group treated with saline [47]. Interestingly, the above three raw materials were also reported to own the fatigue-relieving function [48], and they were used as the positive control in some anti-fatigue experiments.

#### 3.3.2. Effects of SWCC on Exercise Capacity of Mice

**Figure 4:**
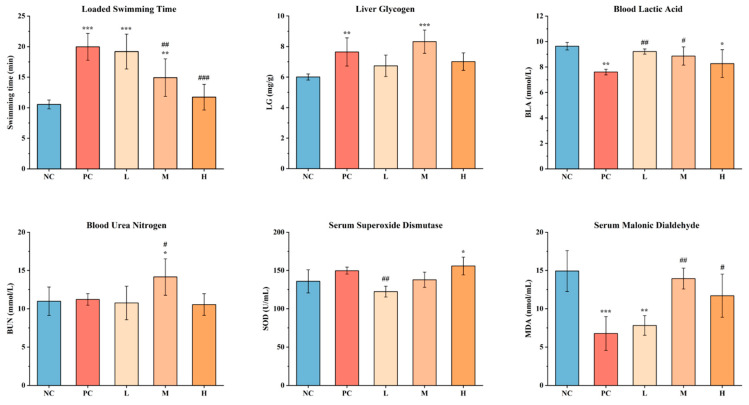
Effect of SWCC treatment on athletic and biochemical indexes related to fatigue. Note: * *p* < 0.05, ** *p* < 0.01, and *** *p* < 0.001: considered statistically significant compared with NC group. # *p* < 0.05, ## *p* < 0.01, and ### *p* < 0.01: considered statistically significant compared with PC group.

Improving athletic endurance is a direct measure of enhanced anti-fatigue capacity, and the loaded swimming time of mice effectively reflects their fatigue state during exercise [42]. Accordingly, the weight-bearing swimming test is considered a key method to evaluate the anti-fatigue effects of a product. Compared with the NC group (10.54 ± 0.73 min), the exhaustive swimming time in the L group (19.19 ± 2.85 min) and M group (14.93 ± 3.07 min) was significantly prolonged (*p* < 0.01), while the H group (11.73 ± 2.09 min) did not show a significant difference (*p* > 0.05), suggesting a negative correlation between SWCC dosage and its effect on prolonging loaded swimming time. Compared with the PC group (19.96 ± 2.19 min), there was no significant difference in the L group (*p* > 0.05), and the M and H groups had significantly shorter times (*p* < 0.01), indicating that both SWCC and ordinary coffee can enhance the exercise capacity of mice. The effects of relieving physical fatigue at the same dose were comparable (Figure 4).

#### 3.3.3. Effects of SWCC on Energy Metabolism in Mice

According to the theory of energy consumption, the decrease in glycogen content during exercise is a primary cause of fatigue [49,50]. During intense physical activity, adenosine triphosphate (ATP) and glucose in cells are rapidly consumed, leading to a decrease in blood glucose levels. Glycogen, as an essential energy substrate, can be converted directly or indirectly into glucose to maintain blood glucose within the physiological range, and can directly produce ATP, which supplies energy during aerobic or anaerobic conditions, thereby regulating energy metabolism [42,51]. Therefore, glycogen levels serve as a critical indicator for assessing the degree of physical fatigue, and changes in glycogen content directly reflect the body’s energy reserves during fatigue. Compared with the NC group (6.01 ± 0.20 mg·g^−1^), liver glycogen (LG) content in the PC group increased to 7.64 ± 0.92 mg·g^−1^ (*p* < 0.01), and in the M group, it increased to 8.32 ± 0.76 mg·g^−1^ (*p* < 0.001). However, there were no significant differences between the SWCC dose groups and the PC group (*p* > 0.05) (Figure 4). The results showed that intragastric administration of SWCC increased liver glycogen storage, enhanced exercise endurance, and alleviated physical fatigue, with the M group showing the most pronounced effects.

#### 3.3.4. Effects of SWCC on the Metabolite Accumulation in Mice

The theory of metabolite accumulation in fatigue suggests that strenuous exercise leads to the production of substantial amounts of metabolites, such as lactic acid and urea nitrogen, which interfere with normal metabolic processes, thus reducing exercise capacity and causing physical fatigue [42,52]. Lactic acid, a byproduct of anaerobic respiration in skeletal muscle cells, accumulates and lowers the pH in muscles and blood, reducing muscle-contraction ability and inducing fatigue [53]. Therefore, blood lactic acid (BLA) levels are a key indicator for evaluating the body’s fatigue status—higher BLA levels correlate with lower exercise capacity and greater fatigue [54]. As shown in Figure 4, compared with the NC group (9.63 ± 0.30 mmol·L^−1^), BLA levels in the PC group decreased to 7.60 ± 0.23 mmol·L^−1^ (*p* < 0.01) and to 8.27 ± 1.09 mmol·L^−1^ in the H group (*p* < 0.05), with no significant difference in anti-fatigue effects between these two groups (*p* > 0.05). However, BLA levels in the L and M groups after unloaded swimming were significantly lower than those in the PC group (*p* < 0.05), indicating that the anti-fatigue effect of SWCC was dose-dependent.

Blood urea nitrogen (BUN) is another indicator of metabolite accumulation. When energy from sugars and fats is insufficient during vigorous activity, proteins undergo metabolic hydrolysis, releasing energy to compensate for the deficit [37]. Thus, BUN levels can indicate the degree of physical fatigue; lower BUN levels suggest reduced protein consumption and improved exercise tolerance [51]. As shown in Figure 4, there were no significant differences in BUN levels among the NC (10.98 ± 1.86 mmol·L^−1^), PC (10.22 ± 0.76 mmol·L^−1^), L (10.76 ± 2.18 mmol·L^−1^), and H (10.55 ± 1.41 mmol·L^−1^) groups (*p* > 0.05), indicating that neither ordinary coffee nor SWCC reduced BUN levels. When the body cannot meet energy demands solely through the catabolism of sugars and fats during high-intensity exercise, proteins are activated as an energy source and undergo decomposition to be converted into glucose, providing energy for muscle activity. In this experiment, given the actual conditions of the mice in the swimming pool, they were forced to swim without a load for only 30 min before blood collection. This exercise intensity may not have been sufficient to trigger protein catabolism, resulting in no significant differences in BUN levels across groups. Additionally, ICR mice treated with ginsenoside (100 mg·kg^−1^·d^−1^) or ginsenoside/Lycium barbarum polysaccharide (2/1) group (100 mg·kg^−1^·d^−1^) for 4 weeks did not perform significant difference in BUN levels with control group with normal saline [4], and there were no significant difference in serum BUN levels between the sea buckthorn seed oil high-dose group (3.35 g/kg·BW) and normal control group with saline [53], indicating that the type or dose of the tested substance may also affect the content of BUN, showing negative results.

#### 3.3.5. Effects of SWCC on Oxidative Stress in Mice

The theory of free radical-induced fatigue suggests that high-intensity exercise enhances oxidative metabolism in the body, and the resulting free radicals cannot be eliminated promptly [53]. This imbalance in free radical metabolism leads to excessive levels of reactive oxygen species, causing damage to cells, tissues, and organs. The body enters a state of oxidative stress, greatly promoting fatigue, which is closely related to the body’s antioxidant capacity [54]. Oxidative stress biomarkers include specific and non-specific antioxidants, lipid peroxidation products, protein oxidation products, and others. SOD is a key antioxidant enzyme that catalyzes the conversion of superoxide anion free radicals (O_2_ˉ) into hydrogen peroxide (H_2_O_2_), which is further converted into water (H_2_O) by antioxidant enzymes such as glutathione peroxidase (GSH-Px) and catalase, thereby effectively mitigating the potential damage of O_2_ˉ to cells [25]. Therefore, increasing SOD levels has a positive effect on alleviating fatigue. Additionally, MDA is a key product of cell membrane lipid peroxidation, and its levels can serve as an indicator to indirectly evaluate the degree of lipid peroxidation [51]. Strenuous exercise induces significant lipid peroxidation, disrupts cellular membranes, and damages mitochondria, ultimately leading to fatigue. Therefore, MDA levels are positively correlated with the degree of fatigue [55].

As shown in Figure 4, the SOD activity in the H group (155.81 ± 11.66 U·mL^−1^) was significantly higher than in the NC group (135.77 ± 15.06 U·mL^−1^) (*p* < 0.05), but there were no significant differences among the PC, M, and H groups (*p* > 0.05). The results demonstrated that SWCC can improve SOD activity in serum and relieve physical fatigue by maintaining the balance between oxidation and antioxidant systems, though a higher intake dose is required for significant anti-fatigue effects. Compared with the NC group (14.92 ± 2.68 nmol·mL^−1^), MDA levels in the PC group were significantly reduced to 6.78 ± 2.21 nmol·mL^−1^ (*p* < 0.001) and to 7.82 ± 1.28 nmol·mL^−1^ in the L group (*p* < 0.01), with no significant difference in the effects between these two groups on lowering MDA levels (*p* > 0.05). The results indicate that both ordinary coffee and SWCC effectively reduce MDA levels, inhibit lipid peroxidation, lessen oxidative stress injury, and alleviate fatigue. Notably, in contrast to the evaluation of in vitro antioxidant capacity using ethanol extraction, the *in vivo* experiment verified its antioxidant properties through two biomarkers (SOD and MDA) by employing hot-water brewing, which simulated real-world consumption scenarios. Two methods were mutually complementary, collectively providing a holistic understanding of SWCC’s antioxidant properties from both a chemical compositional perspective and a biologically functional standpoint.

#### 3.3.6. Comprehensive Analysis of Physiological and Biochemical Indexes

According to the test standards for anti-fatigue products outlined in the *Technical Guidelines for Functional Testing and Evaluation of Health Foods* (2023 Edition), a test sample is considered to have a fatigue-relieving effect if the weight-bearing swimming test shows a positive result and at least two of the three biochemical indicators—BLA, BUN, and LG—are positive. In this experiment, using distilled water in the NC group as a blank control, at least one dose group of SWCC-treated mice exhibited a significantly prolonged load-bearing swimming time (*p* < 0.05), decreased BLA levels (*p* < 0.05), and increased LG levels (*p* < 0.05), while no significant changes in BUN levels were observed across all dose groups (*p* > 0.05). Therefore, considering the positive results of the weight-bearing swimming test and two biochemical indicators, SWCC can be considered to have fatigue-alleviating effects complying with functional food regulations. Notably, the high-dose group did not further enhance these benefits, presenting an inverted U-shaped dose–response relationship for key indicators.

This pattern aligned with findings from related studies on plant-derived anti-fatigue agents. Zheng et al. [6] reported that while sea buckthorn leaf aqueous extracts (mainly polyphenols and flavonoids) improved exercise endurance in rats, high doses failed to augment glycogen storage, which was consistent with our observations on LG levels. In contrast, Bi et al. [7] observed a positive dose-dependence with *Lycium ruthenicum* polyphenols, but their study focused on a single-component system, differing from our multi-ingredient SWCC. The threshold effect in our study may arise from interactions between bioactive components in sea buckthorn, wolfberry, and coffee; excessive polyphenols might disrupt redox balance or the regulation of energy metabolism, limiting the anti-fatigue effects at high doses.

**Figure 5:**
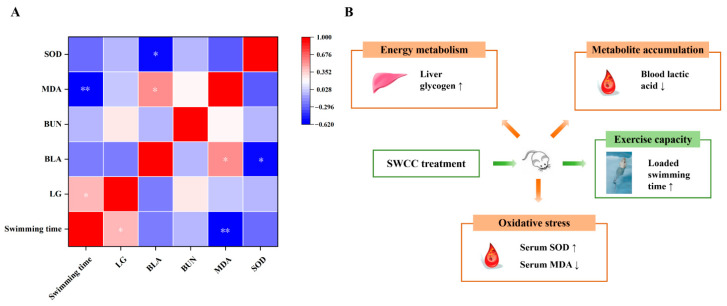
Comprehensive analysis of the anti-fatigue effects of SWCC. (**A**) Pearson correlation analysis between exercise performance, metabolite accumulation, glycogen storage, and oxidative stress. Significant correlations are marked by * *p* < 0.05, and ** *p* < 0.01. (**B**) Overview of the effects of SWCC on the fatigue-related parameters. Note: Upward arrows indicate increased content (elevated levels), and downward arrows indicate decreased content (reduced levels).

Additionally, this experiment investigated the effect of SWCC on antioxidant capacity *in vivo*. The results indicated that, compared to the NC group, at least one of the three SWCC dose groups showed a significant increase in SOD activity (*p* < 0.05) and a significant decrease in MDA levels (*p* < 0.05), suggesting that SWCC’s anti-fatigue effect was related to its antioxidant capacity (Figure 4). Pearson correlation analysis further supports the underlying mechanism (Figure 5A): weight-bearing swimming time was positively correlated with LG levels (r = 0.424; *p* < 0.05) and negatively correlated with MDA levels (r = −0.619; *p* < 0.01). After forced strenuous exercise, BLA levels were negatively correlated with SOD activity (r = −0.560; *p* < 0.05) and positively correlated with MDA levels (r = 0.542; *p* < 0.05). A comprehensive analysis showed that SWCC may regulate energy metabolism and inhibit oxidative stress by increasing glycogen storage, reducing lactic acid accumulation, increasing SOD activity, and reducing MDA levels, thereby improving exercise performance and exhibiting anti-fatigue effects (Figure 5B). Future studies should explore the specific component interactions and molecular mechanisms behind the dose threshold to optimize SWCC’s formulation and application in functional foods [56,57].

## 4. Conclusions

The sea buckthorn–wolfberry compound coffee solid beverage developed in this study enhances the color, flavor, stability, and nutritional quality of conventional coffee products and exhibits significant antioxidant and anti-fatigue effects. The optimized blend of sea buckthorn and wolfberry compound coffee solid beverage was obtained. *In vitro* experiments indicated that SWCC had better antioxidant activity compared to the raw materials of sea buckthorn or wolfberry powder. *In vivo* experiments verified the function of alleviating physical fatigue of SWCC with indexes of significantly longer loaded swimming times, lower blood lactic acid accumulation, and higher liver glycogen reserves compared to distilled water. The locomotor performance of mice had marked relevance to glycogen storage and oxidative stress, and the accumulation of metabolites was significantly correlated with oxidative stress, indicating that the anti-fatigue function of compound coffee was related to antioxidant activity, which may improve the fatigue state of mice and improve the exercise ability of mice by inhibiting oxidative stress. However, SWCC preparation relied on straightforward processes and raw materials produced via singular methods, and its functional evaluation was confined to physical fatigue assessment using forced swimming models. In the future, the types and processing technologies of raw material powders and the preparation processes of the product can be optimized. Furthermore, other nutrients or active ingredients in the product can be investigated, or composite fatigue animal models incorporating physiological and psychological factors can be established, to develop compound coffee products with enhanced properties and functions.

## Figures and Tables

**Table 1:** The orthogonal experiment factor level.

Level	Factor			
	(A) Sea Buckthorn Powder/%	(B) Wolfberry Powder/%	(C) Coffee Powder/%	(D) Additive Mixture/%
1	6	13	59	1
2	9	16	62	2
3	12	19	65	4

**Table 2:** Effect of SWCC on body weight of mice.

Group	Initial Weight/g	1st Weight/g	2nd Weight/g	3rd Weight/g	4th Weight/g	Weight Change/g
NC	31.50 ± 1.86	34.15 ± 2.48	36.07 ± 2.95	36.48 ± 3.30	37.10 ± 3.03	5.60 ± 2.27 ^ab^
PC	31.82 ± 1.43	33.93 ± 1.64	35.14 ± 1.74	35.53 ± 2.05	36.64 ± 2.21	4.82 ± 2.25 ^b^
L	31.93 ± 1.79	34.26 ± 2.43	35.85 ± 2.76	36.89 ± 2.74	37.72 ± 2.92	5.79 ± 1.91 ^ab^
M	31.83 ± 1.70	34.47 ± 1.58	36.29 ± 2.39	37.58 ± 2.28	38.73 ± 2.59	6.89 ± 2.84 ^a^
H	31.24 ± 2.11	33.61 ± 2.12	35.06 ± 2.59	36.10 ± 2.90	37.26 ± 2.69	6.02 ± 1.82 ^ab^

Values in the same column with different letters are significantly different at *p* < 0.05.

**Table 3:** Effects of sea buckthorn–wolfberry compound coffee on mice organs.

Group	Liver Index/%	Kidney Index/%	Spleen Index/%	Heart Index/%	Lung Index/%
NC	4.3 ± 0.38	1.56 ± 0.14	0.22 ± 0.05 ^b^	0.78 ± 0.12	0.70 ± 0.06
PC	3.85 ± 0.57	1.52 ± 0.08	0.20 ± 0.04 ^b^	0.85 ± 0.10	0.78 ± 0.12
L	3.88 ± 0.52	1.54 ± 0.17	0.27 ± 0.04 ^ab^	0.89 ± 0.12	0.83 ± 0.08
M	3.66 ± 0.46	1.44 ± 0.08	0.25 ± 0.09 ^b^	0.86 ± 0.06	0.81 ± 0.08
H	4.30 ± 0.73	1.57 ± 0.16	0.34 ± 0.07 ^a^	0.80 ± 0.16	0.77 ± 0.11

Values in the same column with different letters are significantly different at *p* < 0.05.

## Data Availability

The original contributions presented in the study are included in the article, further inquiries can be directed to the corresponding author.

## Abbreviations

The following abbreviations are used in this manuscript (sorted by the first letter):

ABTS	2,2′-Azino-bis(3-ethylbenzothiazoline-6-sulfonic acid)
BLA	Blood lactic acid
BUN	Blood urea nitrogen
BW	Body weight
DPPH	Free radical scavenger activity on 2,2-diphenyl-1-picrylhydrazy
FRAP	Ferric-reducing antioxidant power
LG	Liver glycogen
MDA	Malondialdehyde
SOD	Superoxide dismutase
SSC	Soluble solids content
SWCC	Sea buckthorn–wolfberry compound coffee
TFC	Total flavonoid content
TPC	Total phenol content
